# Investigating the Quality and Purity Profiles of Olive Oils from Diverse Regions in Selçuk, İzmir

**DOI:** 10.3390/molecules29051104

**Published:** 2024-02-29

**Authors:** Tolga Akcan

**Affiliations:** Food Processing Department, Efes Vocational School, Dokuz Eylül University, 35920 İzmir, Turkey; tolga.akcan@deu.edu.tr

**Keywords:** İzmir, Selçuk, virgin olive oil, quality, geographical indication, phenolic compounds, fatty acid composition, sterols

## Abstract

The Selçuk district of Izmir is one of the most essential regions in terms of olive oil production. In this study, 60 olive oil samples were obtained from five different locations (ES: Eski Şirince Yolu, KK: Kınalı Köprü, AU: Abu Hayat Üst, AA: Abu Hayat Alt, and DB: Değirmen Boğazı) in the Selçuk region of Izmir during two (2019–2020 and 2020–2021) consecutive cropping seasons. Quality indices (free acidity, peroxide value, p-Anisidine value, TOTOX, and spectral absorption at 232 and 270 nm) and the fatty acid, phenolic, and sterol profiles of the samples were determined to analyze the changes in the composition of Selcuk olive oils according to their growing areas. When the quality criteria were analyzed, it was observed that KK had the lowest FFA (0.11% oleic acid, PV (6.66 meq O_2_/kg), p-ANV (11.95 mmol/kg), TOTOX (25.28), and K232 (1.99) values and K270 had the highest value. During the assessment of phenolic profiles, the ES group exhibited the highest concentration of the phenolic compound p-HPEA-EDA (oleocanthal), with a content of 93.58 mg/kg, equivalent to tyrosol. Upon analyzing the fatty acid and sterol composition, it was noted that AU displayed the highest concentration of oleic acid (71.98%) and β-sitosterol (87.65%). PCA analysis illustrated the distinct separation of the samples, revealing significant variations in both sterol and fatty acid methyl ester distributions among oils from different regions. Consequently, it was determined that VOOs originating from the Selçuk region exhibit distinct characteristics based on their geographical locations. Hence, this study holds great promise for the region to realize geographically labeled VOOs.

## 1. Introduction

In the Mediterranean region, Turkey is one of the leading producers of virgin olive oil (VOO), together with Spain, Italy, Greece, Portugal, and other International Olive Council (IOC) member countries, accounting for more than 93% of global production [1]. This prominent position underscores Turkey’s significant contribution to the olive oil market alongside its Mediterranean counterparts. Following in the footsteps of nations renowned for olive cultivation, Turkey exemplifies the region’s rich tradition of olive oil production. From 2011 to 2020, olive grove areas in Turkey experienced an 11.1% expansion, while the number of fruit-bearing trees increased by 35.1% during the same timeframe. The reports indicate that olive oil production in 2022 amounted to 2,037,783 tons. Analyzing olive trees that bore fruit in 2022, it is known that 68.3% of the trees were oil-giving, and 31.7% were table olive trees. Olive cultivation in Turkey is conducted across five distinct regions: the regions of Aegean, Marmara, the Mediterranean and Southeastern Anatolia, and the Black Sea [2]. The Marmara and Aegean regions stand out as the primary areas for olive oil production among these regions. In Turkey, 47.62% of the total olive production area for table olives, and 56.16% for olive oil, is in the Aegean Region [3]. According to the ten-year average, the provinces with the highest olive oil production in Turkey are Aydın, İzmir, Muğla, Balıkesir, and Manisa, respectively. However, based on the data from 2022, the highest output among these provinces is observed in İzmir (410,000 tons) [2]. In the province of Izmir, the Selçuk district comprises 4.31% of the entire agricultural land, with fruit orchards occupying 80% of its agricultural area. The fruit orchard space in Selçuk constitutes 8% of Izmir’s total fruit orchard area. Within this area, 62% of space is allocated for olive oil production [4].

The predominant component of VOO is triacylglycerol, which makes up more than 95% of its content. Simultaneously, the residual portion comprises fewer constituents, including tocopherols, phenols, sterols, hydrocarbons, and volatile compounds [5,6,7]. The chemical and sensory attributes of virgin olive oils vary within defined boundaries due to the absence of a consistent chemical composition profile. The ultimate quality of the oil extracted from the olive mill is influenced by numerous variables, including the type of olive cultivar and its level of ripeness, the agricultural methodologies employed, the harvesting and transportation techniques, as well as the technological procedures and storage conditions utilized during the production of virgin olive oil. While many of these factors are currently under control or subject to modification, the impact of geographical origin, encompassing aspects such as cultivar type, soil composition, and climatic conditions, is emerging as a significant determinant in discerning and safeguarding associated quality standards [8]. When virgin olive oils from identical olive cultivars, but which are cultivated in distinct regions, were analyzed, variations were noted in the sterol, phenolic, and volatile constituents [9,10,11,12]. Due to variations in these factors, producers are making significant efforts to obtain geographical indication (GI) registration for their products, aiming to document the distinctive characteristics of olive oils and ensure the authentication of their unique properties.

To satisfy consumer expectations regarding authenticity and excellence, producers often seek certification of their virgin olive oils based on their geographical indication [13]. The GI is a designation placed on goods from a specific region and highlights attributes or prestige associated with that region. Furthermore, the geographical indication for virgin olive oil helps to protect consumers and producers from economic deceit. It is a helpful method for confirming origin and defending the interests of rural producers [14]. It also serves as a reference for formulating uniform standards for GI virgin olive oils. In this way, it promotes product quality improvement from the point of view of both producers and consumers [15]. The Turkish Patent and Trademark Office and the European Commission implement three certification labels or GI categories: protected designation of origin (PDO) and protected geographical indication (PGI).

Products may also receive certification as a traditional specialty guaranteed (TSG) item [16]. In Turkey and the European Union, certifications such as PDO or PGI are employed for virgin olive oils. There are 23 unique GI products for VOO in Turkey, but only 4 are officially registered in the eAmbrosia EU GI registry [17,18].

In geographical indication applications, it is crucial to identify the chemical characteristics that distinguish one sample of olive oil from others using analytical methods and examining trademarks. Particularly, emphasis has been placed on the distinctive qualities of geographical indications included in the Geographical Indication Registration Database (eAmbrosia) system maintained by the European Commission. In this field, it is observed that during the geographical indication registration applications for Turkish olive oil products, the distinctive characteristics of the products are not thoroughly determined, and factors such as technical difficulties and producers’ concerns regarding constraints are considered among the reasons for this.

This study aims to determine the quality and purity characteristics of olive oils obtained from different regions of the Selçuk district in Izmir, documenting their properties and identifying potential distinctive features. Thus, the findings obtained from our study are expected to contribute to the geographical indication potential of the district’s olive oil, thereby adding to the scientific literature.

## 2. Results and Discussion

### 2.1. Quality Parameters

The quality characteristics of samples of virgin olive oil from five distinct regions within the Selçuk area of Izmir are presented in the following Table 1. One of the most important quality and classification parameters for olive oils is the amount of free fatty acids measured as acidity. It is widely recognized that FFA levels are influenced by numerous factors, such as the fruit quality, timing of harvest, storage conditions, and production processes [19]. When the samples are analyzed, it is seen that FFA values vary between 0.11% (DB) and 0.27% (AA). Regional differences were observed to have statistically significant effects on FFA values (*p* < 0.05). In line with our research, Öğütçü et al.’s [8] study revealed variations in the acid values of olive oils across different regions within the Çanakkale province of Turkey. Free fatty acid (FFA) content is recognized as the primary criterion for classifying VOO. All samples had acidity levels below the maximum legal threshold of 0.8% for Extra Virgin Olive Oil (EVOO), as measured using the percentage of oleic acid [20]. Öğütçü et al. [8] noted that traditionally, consumers favor olive oils with acidity levels of 1% or lower. Our findings also indicate that this preference holds for consumers. The values found are lower than the acidity values of South Aegean olive oil with geographical marks, especially Milas olive oil, which is geographically registered by the European Union [16,21]. PV and panV are quality indicators that indicate the extent of oxidation in oil, offering insights into its oxidative degradation and estimated storage duration. When the samples were analyzed, a statistical difference was found between the regions regarding their PV values. The ES group had the highest value of 11.69 (meq O_2_/kg), while the DB group had the lowest value of 6.76 (meq O_2_/kg) (*p* < 0.05). There was no statistically significant difference between AU and AA groups (*p* > 0.05). Elevated peroxide index levels are likely due to prolonged exposure of the olives before oil extraction or increased damage between harvesting and processing [22]. The peroxide index establishes the degree of initial oxidation, the reaction of rancidification in olive oil, and the potential degradation that has taken place in naturally occurring antioxidants, primarily tocopherols and polyphenols [23]. Remarkably, all values fell beneath the threshold of 20 meqO_2_/kg oil, as stipulated by the International Olive Council [20], which serves as the benchmark for categorizing EVOO. p-anV is a value reflecting the amount of aldehydes from secondary oxidation products, and values of 11.95–24.08 were observed in KK and DB groups, respectively. Statistically significant differences were found between the groups (*p* < 0.05). In parallel with the PV and pANV values, the TOTOX values of the groups were similar. The highest and lowest values were found in the ES (47.41) and KK (25.28) groups, respectively (*p* < 0.05). The values found in our study were found to be lower than the TOTOX data obtained in the study in which olive oils with geographical indications in Turkey were examined [16]. Contrary to our study, no statistically significant differences were found between the samples in the same study. In general, the absorption level at 232 nm demonstrates an inclination to elevate in correlation with the extent of diene conjugation and the existence of primary oxidation byproducts, while absorption at 270 nm shows augmentation in correspondence with triene conjugation and the occurrence of secondary oxidation byproducts. Moreover, both assessments have been employed to ascertain the adulteration of virgin samples with refined oils. Typically, the incorporation of refined oils tends to elevate both values [24]. In accordance with the standards set by the IOC, the K232 and K270 values must not exceed 2.50 and 0.22, respectively, for classification as EVOO. The highest K232 value was observed in the DB (2.25) group and the lowest value was observed in the AU (1.98) group. Elevated K232 readings in these samples could suggest inadequate storage conditions for the oils. The minimum and maximum k270 values were 0.11 (DB) and 0.14 (KK), respectively. In the study conducted by Sevim et al. [21], K232 and K270 values of olive oils obtained in the 2014 and 2015 seasons show similar values with our study. The oils obtained from Memecik-type olives used in oil extraction in the Selçuk region were observed to have similar values. Comparable findings to our results were also identified in analogous studies documented in the literature [25,26,27]. Factors such as fruit health, storage conditions, harvesting methods, and transport can affect the quality parameters of oils, while geographical origin is reported to have an insignificant effect [28,29]. However, our research contradicts this idea. Consistent with our findings, samples collected from various regions within the same province or different provinces were reported to exhibit differences in quality characteristics [8,16,21,30].

### 2.2. Phenolic Profiles of Samples

During two consecutive seasons, we examined the total and individual amounts of eight phenolic compounds in olive oils sourced from five distinct areas in the Selçuk district of Izmir (Table 2). The amounts of each phenol were added to determine the total phenol contents. The phenolic composition undergoes alterations as the olives mature [31]. In contrast to the study conducted by Boussahel et al. [30], oil samples showed statistically significant differences in terms of phenolic composition, although they were of a single variety (*p* < 0.05). Various phenolic compounds have been detected in Turkish virgin olive oils across different studies documented in the literature. Türkay et al. [16] showed that the concentration of *p*-coumaric acid in commercial extra virgin olive oils obtained from the Aegean region varied between 2.2 and 4.8 mg/kg. A prior investigation reported that the concentration of *p*-coumaric acid in commercial extra virgin olive oils obtained from the Aegean region varied between 0.10 and 0.69 mg/kg [5,32]. Pinoresinol, recognized by numerous scientific studies as the characteristic lignan present in virgin olive oils, was conspicuously absent from specific studies of the phenolic composition of these oils. This absence can be attributed to two possible factors. Firstly, minor ambiguities within chromatographic analyses could lead to misidentifications. Secondly, the phenolic profile of virgin olive oils is substantially influenced by seasonal, regional, and varietal variations, as proposed by [5,10,16,33]. Specifically, *p*-HPEA-EDA, also known as oleocanthal, was identified as the predominant compound within the sample. The concentrations of phenolic compounds in the samples showed differences across the AA (107.92 mg/kg), KK (183.99 mg/kg), DB (202.12 mg/kg), ES (219.66 mg/kg), and AU (226.88 mg/kg) groups. The quantities of 3,4-DHPEA present in virgin olive oil samples ranged from 1.55 (ES) to 11.53 (DB) mg/kg (*p* < 0.05). The p-HPEA value was highest in the AU (18.67 mg/kg) samples (*p* < 0.05). In our samples, the concentration of *p*-coumaric acid ranged from 0.95 to 1.18 mg/kg, with the AU group exhibiting the highest *p*-coumaric acid content (*p* < 0.05). The ES group had the highest pinoresinol content, measuring 18.31 mg/kg (*p* < 0.05). The primary phenolic compound detected was *p*-HPEA-EDA (oleocanthal), with concentrations ranging from 42.43 (AA) mg/kg to 93.58 (ES) mg/kg. Additionally, 3,4-DHPEA-EA (oleuropein aglycon monoaldehyde) was present in quantities ranging from 25.24 (KK) mg/kg to 40.44 (AU) mg/kg, while 3,4-DHPEA-EDA (oleacein) ranged from 6.87 (AA) mg/kg to 55.95 (DB) mg/kg. In the ES group, *p*-HPEA-EA (lignostride aglycone monoaldehyde) exhibited the highest concentration at 15.21 mg/kg; in the AA group, it displayed the lowest concentration at 6.50 mg/kg. Yorulmaz et al. [32] observed minimal variability among individual phenolics in oils derived from the same cultivar but grown in distinct regions. They noted a significant impact of the variety; however, they also identified variations in luteolin contents and phenol concentrations of the same variety across different regions, a finding consistent with our study. Similarly, the oleocanthal values reported in Türkay et al.’s [16] studies on South Aegean olive oils of the Memecik variety, which we incorporated in our research, align with our findings. Additionally, Türkay et al. [16] noted lower 3,4-DHPEA-EA values than those identified in our study. This observation suggests that this value could serve as a distinguishing characteristic of the Selçuk region.

**Figure 1:**
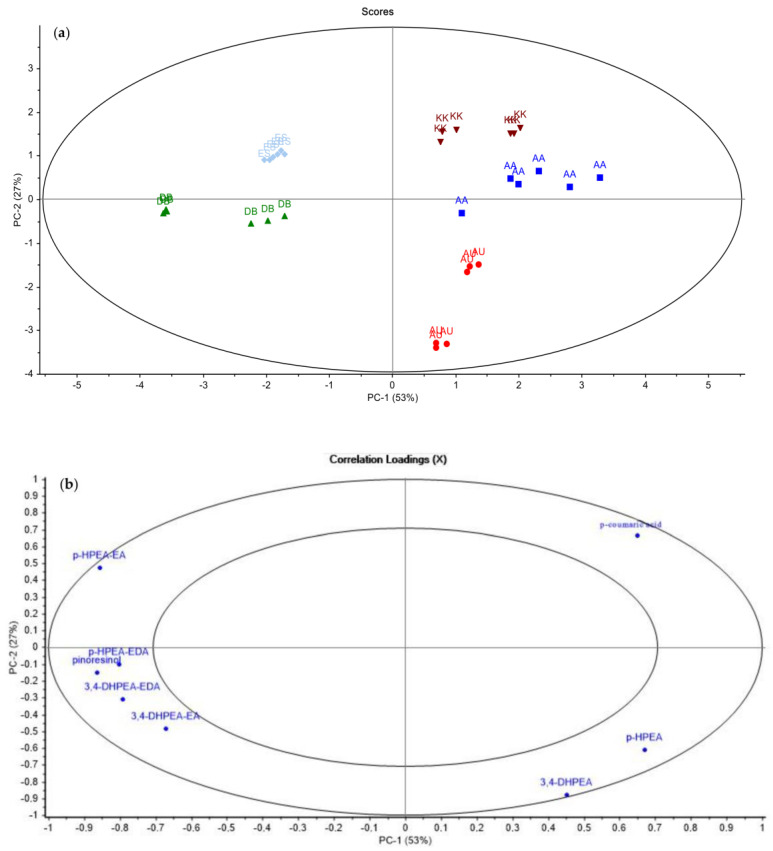
PCA score (**a**) and loading plots (**b**) for phenolic composition data.

The initial two principal components derived from the PCA analysis utilizing phenol composition data elucidated a cumulative variance of 80%, accounting for the dissimilarities observed among the samples. This was depicted in the score plot (Figure 1a). In the score plot (Figure 1a), it can be seen that the oils of the KK, AA, and AU regions are easily separated from the other sample groups on the positive side of the PC1 axis. When the distributions of the variables in the loading plot of PCA were analyzed, it was seen that this separation was due to simple phenols. In particular, it was observed that QC samples were clustered in the positive region of PC1 and PC2, and it is thought that *p*-coumaric acid is effective. As seen in Table 2, the coumaric acid content of the KK group was found to be significantly higher than the other groups (*p* < 0.05). Similar to these results, Korkmaz [34] reported that regional differences in phenolics of different varieties from olive oils obtained from different regions of Turkey appeared to be distinctive in PCA analysis. ES samples were clustered in a different position from the other sample groups in the score plot, with PC2 in the positive and PC1 in the negative part of the score plot. This is thought to be the effect of *p*-HPEA-EA. The results shown in Table 2 support this conclusion.

### 2.3. Fatty Acid Composition

Concerning the fatty acid (FA) profile, VOO samples exhibited 12 distinct fatty acids, detailed in Table 3, which comprehensively depicts the oil’s composition. The primary fatty acids identified within our groups included oleic, linoleic, palmitic, and stearic acids. In our study, oleic acid was found to be between 66.92% (KK) and 71.98% (AU) (*p* < 0.05). The samples’ oleic acid content complied with the standards set by the IOC (2003). Köseoğlu et al. [26] observed a reduction in the oleic acid content of olive oils as the skin color of olives transitioned from green to black. The variations among our groups are believed to stem from differences in their maturation levels, with the maturation process potentially occurring faster in the KK region. Palmitic acid ranged from 13.09% to 14.42% across all groups. Both the ES and KK groups exhibit palmitic acid concentrations exceeding 14% (*p* < 0.05). The concentration of stearic acid (C18:0), a noteworthy fatty acid in virgin olive oils, ranged from 2.08% (ES) to 2.56% (AA). The linoleic acid values were found to vary between 8.01% (AA) and 10.57% (ES) after analysis, within the limits established as 2.5–21.0% by IOC (*p* < 0.05). Similarly, the levels of linolenic acid in the olive oil samples in our study, ranging between 0.59% and 0.69%, stayed below the limit established by the IOC (1.0%). Statistically significant differences were noted in the behenic acid values among the results, particularly with the KK (0.10%) and AU (0.11%) groups exhibiting lower levels than the other groups. Regarding MUFA values, there was no statistically significant variation between the samples (69.90–73.36%) (*p* > 0.05). The samples’ PUFA values showed that the ES group had the highest content (11.26%), and the AA group had the lowest concentration (8.66%) (*p* < 0.05). The AU group exhibited the lowest saturated fatty acid (SAFA) rate at 15.90% (*p* < 0.05). Similar to our investigation, another study found a substantial correlation between the fatty acids in olive oils derived from the “Ayvalık” variety and the region in which they were grown [35]. Gürdeniz et al. [36] discovered that virgin olive oil from the Memecik variety in the İzmir region had an oleic acid content of approximately 71.2%, consistent with our findings. In a separate investigation, where the fatty acid compositions of oils from the South Aegean region were analyzed, the outcomes mirrored those obtained in our study [16,37]. These findings are in contrast to another study that revealed a lower oleic acid concentration (65%) in an investigation on the characteristics of arbequina-variety EVOOs cultivated in the Aegean region [38].

**Figure 2:**
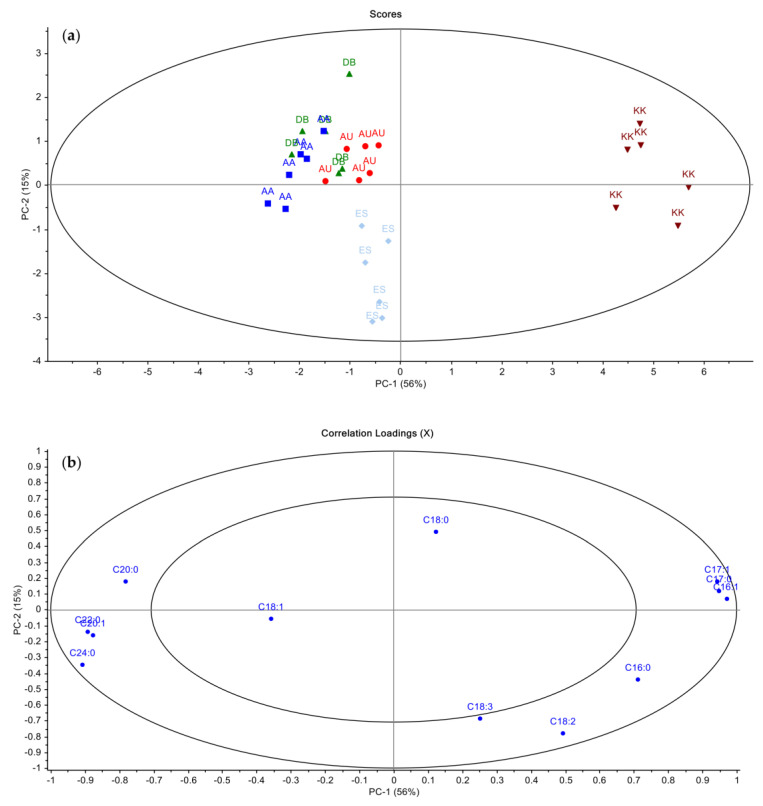
PCA score (**a**) and loading plots (**b**) for fatty acid composition data.

The first two principal components obtained as a result of the PCA analysis with fatty acid composition values explained 71% of the differences between the samples in total. In the score plot (Figure 2a), the KK samples are easily separated from the other sample groups on the positive side of the PC1 axis. According to the distributions of variables in the loading plot of PCA (Figure 2b), it is seen that the samples are located on the positive side of PC1 due to the high amounts of C17:0, C17:1, and C16:1. Similarly, ES samples were clustered on the negative side of PC2 and PC1 in the score plot and at a different location from the other sample groups. Although the olive groves are located at distances that do not allow significant climatic variations, differences in the fatty acid composition of VOOs determined using chemometric methods were detected. The main reason is microclimatic differences such as wind regime and sunlight exposure time within the selected region.

Previous research on distinguishing VOOs from closely situated regions concluded that utilizing fatty acid composition data with chemometric techniques did not lead to practical separation. In a previous study, the weak grouping of VOO samples in the PCA score plot was observed when employing fatty acid composition data for the Mediterranean and southeastern regions, which are geographically close [39]. In a similar context, Kritioti et al. [40] emphasized that relying solely on fatty acid composition did not offer sufficient information for a hierarchical cluster analysis in classifying VOOs from the southern region of Cyprus, given the relatively small total area. The researchers suggested that the limited climatic diversity did not significantly impact the fatty acid composition [40]. Likewise, Karabagias et al. [41] conducted a study sampling VOOs from four different Greek islands in the Ionian Sea, utilizing chromatographic data to create canonical discriminant functions for geographical origin discrimination. Notably, only VOOs from Corfu island could be differentiated from those of the Lefkada, Kefalonia, and the Zakynthos islands, as the latter three were in closer proximity, and at the same time, Corfu was situated at a greater distance from them [41]. These findings align with the argument presented by Kritati et al. [40] that achieving regional discrimination for small sampling areas solely using chromatographic data and chemometric techniques is challenging.

### 2.4. Sterol Composition

Sterol content and composition are pivotal factors influencing the quality of extra-virgin olive oil, particularly regarding its nutritional value. Additionally, these compounds serve as crucial metrics for adhering to commercial standards, verifying the authenticity of olive oil, and detecting counterfeit products. The alteration caused by hydrolytic and oxidative processes can impact the fatty acid composition. Consequently, sterols offer significant potential as valuable chemical markers for discerning olive oil’s variety and geographic sources and maintaining its traceability [42]. Table 4 shows the sterol contents of olive oils obtained from five different regions of the Selçuk district. The highest cholesterol content was found in the AU group, at 0.15%. It was observed that the campesterol contents of the groups varied between 3.09% (DB) and 3.34% (KK). Stigmasterol values were found to vary between 1.36 (KK) and 1.83% (DB). Δ7-stigmastenol values were between 0.20 (AU) and 0.40% (AA). All these values were found to be below IOC standards [43]. In a study on olive oil quality of different cities in Algeria, it was found that city differences had effects on sterol composition [44]. According to the Codex Alimentarius, apparent β-sitosterol, encompassing Δ5.23-stigmastadienol, clerosterol, β-sitosterol, sitostanol, Δ5-avenasterol, and Δ5.24-stigmastadienol, must exceed 93% in virgin olive oil. Our study revealed values exceeding the specified threshold in the Codex Alimentarius. Phenolic compounds and vitamin E are the most crucial factors in preserving olive oil [45,46]. In addition, it is stated in the studies that β-sitosterol provides significant protection [47,48]. The Δ-5-avenasterol levels exhibited variation, ranging from 3.47% (DB) to 5.21% (ES). Notably, Δ-5-avenasterol is recognized as an antioxidant and anti-polymerization agent in frying oils [49,50].

**Figure 3:**
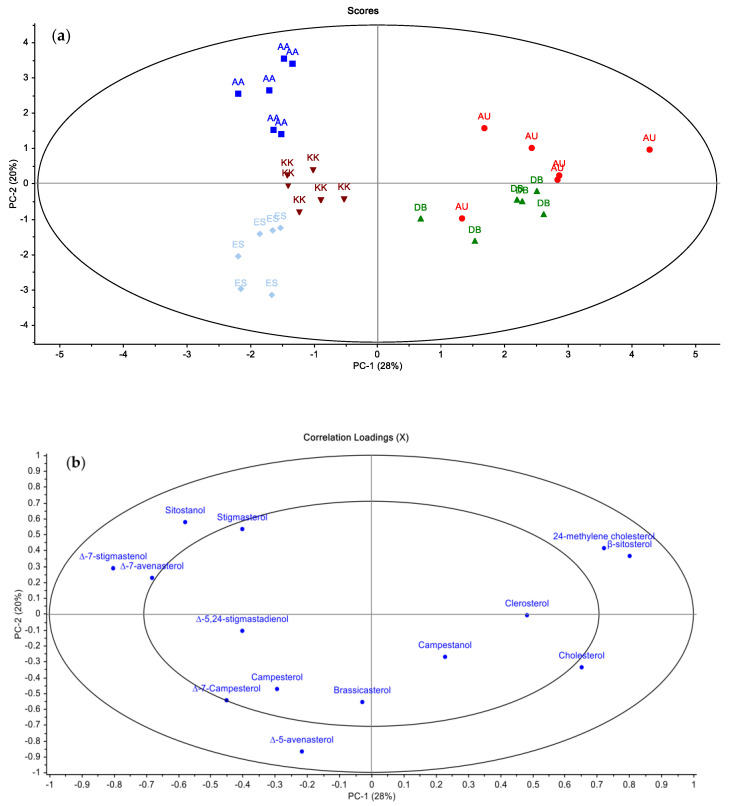
PCA score (**a**) and loading plots (**b**) for sterol composition data.

Sterol composition reliably assists in the classification of olive oil. The data depicted in Figure 3 demonstrate a discernible pattern where the score and loading plots reveal the existence of two primary clusters based on PC1. Locations AU and DB exhibited distinct characteristics that set them apart from the other locations (Figure 3a). The observed disparity is likely attributable to the variations in altitude between the locations. Conversely, PC2 offers additional differentiation among the groupings ranging from AA to ES. In line with our investigation, it was noted that the sterol composition of olive oils sourced from various provinces in Turkey varied according to geographical factors [51]. Upon completion of PCA, the power analysis of the modeling results revealed that β-sitosterol, Δ5-avenasterol, Δ7-campesterol, Δ-7-avenasterol, campesterol, Δ-7-stigmastenol, sitostanol, and 24-methylene cholesterol are the most influential variables in distinguishing between olive oils, as evidenced by the loading plot depicted in Figure 3b. As in the fatty acid composition, in the sterol composition, ES samples were clustered on the negative side of PC2 and PC1 in the score plot and at a different position from the other sample groups. Sitostanol and stigmasterol contents were found to be essential variables in characterizing the olive oil samples collected from the AA region, and campesterol and Δ7-campesterol emerged as particularly significant variables for characterizing the olive oils from ES. Typically, it can be concluded that the sterol compositions of β-sitosterol, Δ-5-avenasterol, campesterol, cholesterol, stigmasterol, clerosterol, and Δ-7-stigmastenol were utilized as the primary components in olive oil used to verify the authenticity of PDO olive oil and to characterize various olive oils, aligning with the existing literature [52].

## 3. Materials and Methods

### 3.1. Sampling Virgin Olive Oil Products

**Figure 4:**
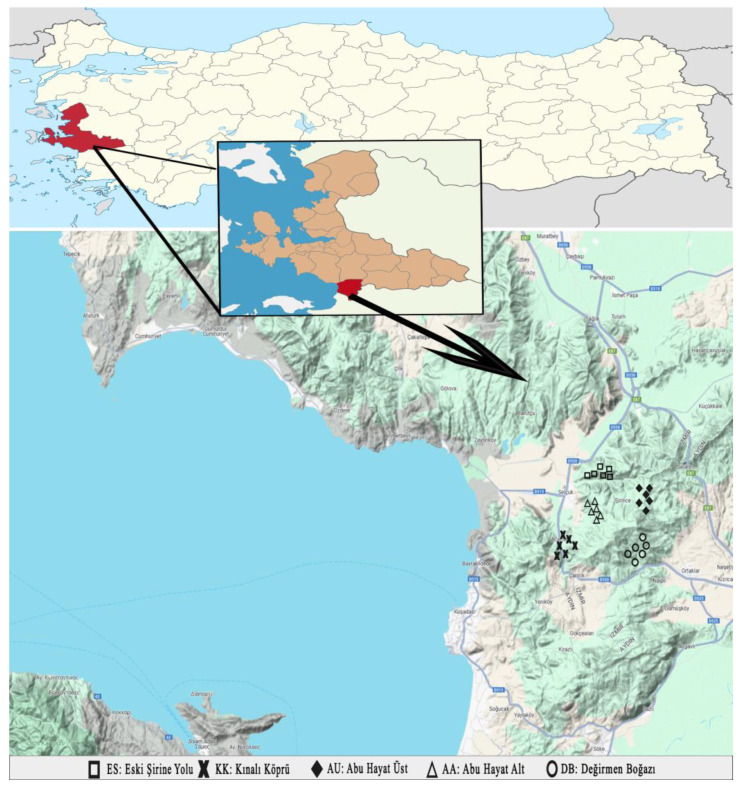
Map of olive oils collected from 5 different regions of Selcuk, İzmir.

Sixty monovarietal VOO samples obtained from Memecik olive cultivars representing the 2019–2020 and 2020–2021 harvest seasons from 5 different regions (ES: Eski Şirince Yolu, KK: Kınalı Köprü, AU: Abu Hayat Üst, AA: Abu Hayat Alt, and DB: Değirmen Boğazı) of the Selçuk district were obtained from the Selçuk Chamber of Commerce (6 orchards × 5 districts × 2 crop seasons). Twelve samples were collected over two seasons, with six samples collected from each region during each season. In order to reduce the seasonal effect only in PCA models, our models were created by randomly selecting six samples from a total of twelve samples from each region. Thus, PCA models were created for 30 samples from 5 regions. The geographic coordinates of each olive oil group from different regions are shown in Figure 4—all samples were produced in dual-phase centrifugation systems. After filtration, the VOO samples were poured into glass amber bottles, and the headspace was purged with nitrogen before sealing the lids. The samples were then placed in a freezer at −24 °C for subsequent analysis.

### 3.2. Reagents

A mixture of standard fatty acid methyl esters (FAMEs), standards of syringic, tyrosol, and 5α-cholestan-3β-ol were sourced from Sigma-Aldrich (Steinheim, Germany). Diethyl ether, acetic acid (glacial), chloroform, and methanol were obtained from ISOLAB (Eschau, Germany). Sodium hydroxide, sodium carbonate, sodium thiosulfate, phosphoric acid, and ethanol were purchased from Fluka (Buchs, Switzerland). p-Anisidine, cyclohexane, and acetonitrile were obtained from Merck (Darmstadt, Germany).

### 3.3. Determination of Quality Parameters

The FFA (in oleic acid %) [53] and UV spectrophotometric indices (K232 and K270 measurements) were measured according to the methods given by the IOC [54].

### 3.4. Peroxide Value (PV) Analysis

The samples’ peroxide values (PV) were determined employing the COI/T.20/Doc. No 35/Rev.1 method [55]. The results were expressed as milliequivalents of active oxygen per kilogram of oil (meq O_2_/kg sample).

### 3.5. p-Anisidine Value (p-anV) Analysis

The p-anisidine value was assessed following the AOCS-Cd-18 90 method. The outcomes were expressed in mmol/kg oil [56].

### 3.6. Total Oxidation Value (TOTOX)

TOTOX is an indicator of oil degradation and was computed using the equation: TOTOX = 2PV + p-anV [56].

### 3.7. Determination of Methyl Esters of Fatty Acids

The examination of the fatty acid composition in the samples was conducted through a gas chromatography system (Nexis GC-2030, Shimadzu, Japan) equipped with a flame ionization detector (FID), as outlined in the IOC methods [57]. Fatty acid methyl esters were prepared by dissolving 0.1 g of oil sample in 5 mL of *n*-hexane and 1 mL of potassium hydroxide with methanol. Rt-2560, 100 m, 0.25 mm ID, and 0.20 µm capillary column (Restek Columns) was used for analyses. The injection volume was one μL, and the temperature of the detector and injector was 250 °C. The oven temperature was programmed from 170 to 210 °C in increments of 2 °C/min. The analysis was terminated by keeping the temperature at 210 °C for 10 min. The Supelco FAME mix was used as a reference standard to identify the fatty acids of the olive oil samples. The Shimadzu Lab solutions program calculated all fatty acid peak areas and recorded the peak area percentage.

### 3.8. Phenolic Profile Determination

Minor phenolic compounds with polar characteristics were extracted and subsequently quantified using high-pressure liquid chromatography (HPLC) according to the standard method of the IOC [58]. Some modifications were made based on Türkay et al. [16] to improve accuracy. HPLC (Agilent 1260 model infinity II, Santa Clara, CA, USA) was equipped with a Spherisorb ODS-2 C18 reverse-phase column (4.6 mm × 25 cm), a 100 A° spectrophotometric UV detector, and an integrator at 280 nm. Two grams of the sample were carefully weighed and transferred into glass tubes, after which 1 mL of a solution containing syringic acid was introduced as an internal standard. The resulting mixture underwent vortexing for 30 s, and then 5 mL of a methanol and water solution in an 80/20 (*v*/*v*) ratio was added. Subsequently, the samples underwent a 15 min ultrasonic treatment in a bath, followed by centrifugation at 5000 × rpm for 25 min. The injection volume was 40 μL. The mobile phases were water (0.2% H3PO4 *v*/*v*) (A), methanol (B), and acetonitrile (C), and the flow rate was 0.8 mL/min with a gradient flow composition. The gradient elution was as follows, starting from 96% A, 2% B, and 2% C; 50% A, 25% B, and 25% C at 40 min; 40%, 30%, and 30% at 5 min; 50% B and 50% C at 15 min with a 10 min standby; and 96% A, 2% B, and 2% C at 2 min with 10 min standby. The peaks were identified according to the relative retention time of the internal standard peak (syringic acid) described in the standard method. To quantify and express the identified phenolics as tyrosol, the respective response factor (RRF value) was found to be 4.7 (the ratio of the response factor of syringic acid to tyrosol), as described in the standard method. The peaks were identified using the RRT of phenolic compounds against syringic acid, which served as the internal standard of the method.

### 3.9. Determination of Sterol Composition

The method described by Karacan et al. [59] was used to determine the sterol composition of the samples. Diethyl ether was employed to extract the unsaponifiable portion of olive oil, while thin-layer chromatography isolated the unsaponifiable sterol fraction. The analysis was performed using Gas Chromatography (Nexis GC-2030, Shimadzu, Japan) equipped with a flame ionization detector (FID) with a 30 m × 0.25 mm × 0.25 μm Rxi-SVOCms column. The injection volume was one μL, and the detector’s temperature was 290 °C. The temperature of the furnace was programmed from 260 °C. Helium was used as carrier gas at a 0.5 mL/min injection volume. The flow rate was 0.9 mL/min. The split ratio was 1:40. It was silylated using pyridine and Bis(trimethylsilyl) trifluoroacetamide and trimethylchlorosilane as the silylation reagent. 5α-cholestan-3β-ol was used as the internal standard in the analysis. Peak areas were expressed as a percentage.

### 3.10. Statistical Analysis

To improve the accuracy of the results, three measurements and duplicate analyses were carried out. An analysis of variance (ANOVA) followed by Tukey’s post hoc test, with a significance level set at 5% (*p* < 0.05), was employed using IBM SPSS 26 to assess disparities in quality attributes among samples of VOO. Unscrambler X (Camo Analyt-ics, Oslo, Norway) was used to perform two principal component analyses in order to visually portray probable discriminations among VOO samples. The data on the composition of fatty acids and sterols were randomized, mean-centered, and internally validated using the leverage correlation approach. All variables were weighted as the reciprocal of the standard deviation to remove the impact of size differences on the outcomes. The singular value decomposition (SVD) approach was chosen to construct the PCA model. There was no rotation used. Two-dimensional biplots were generated to visualize the first two principal components (PCs). Discrimination was determined using the score plots.

## 4. Conclusions

This study established a significant data set, including quality indices and the fatty acid and sterol composition of VOOs of five different regions of the Selçuk district of Izmir. The primary fatty acids are oleic, palmitic, linoleic, stearic, and palmitoleic. The fatty acids, quality criteria, and sterol compositions isolated from the obtained oils strongly correlated with the regions from which they were obtained. A PCA analysis illustrated the distinct separation of the samples, revealing significant variations in both sterol and fatty acid methyl ester distributions among oils from different regions. Notably, a more pronounced clustering pattern was observed in the distribution of fatty acids. It is thought that microclimatic differences such as wind regime and sunlight exposure time in the region and altitude may be effective. Therefore, considering the geographical differences, the region seems to have significant potential for obtaining new geographical registrations, which is one of the most competitive economic strategies for value-added VOOs.

## Figures and Tables

**Table 1:** Quality characteristics of virgin olive oils from five different locations in the Selçuk region.

Groups	Free Fatty Acid Content (% Oleic Acid)	Peroxide Value (meq O_2_/kg)	*p*-Anisidine Value (mmol/kg)	Total Oxidation Value	K232	K270
ES	0.13 ± 0.01 ^c^	11.69 ± 1.19 ^a^	24.03 ± 0.71 ^b^	47.41 ± 2.39 ^a^	2.19 ± 0.11 ^a^	0.12 ± 0.00 ^c^
KK	0.11 ± 0.01 ^d^	6.66 ± 0.11 ^c^	11.95 ± 0.32 ^d^	25.28 ± 0.53 ^e^	1.99 ± 0.06 ^b^	0.14 ± 0.01 ^a^
AU	0.15 ± 0.01 ^b^	9.73 ± 0.39 ^b^	25.50 ± 0.42 ^a^	44.97 ± 0.94 ^b^	1.98 ± 0.04 ^b^	0.10 ± 0.00 ^d^
AA	0.27 ± 0.01 ^a^	9.77 ± 0.44 ^b^	13.88 ± 0.54 ^c^	33.43 ± 1.24 ^d^	2.21 ± 0.09 ^a^	0.13 ± 0.01 ^b^
DB	0.10 ± 0.01 ^d^	6.76 ± 0.10 ^c^	24.08 ± 0.57 ^b^	37.60 ± 0.74 ^c^	2.25 ± 0.10 ^a^	0.11 ± 0.00 ^cd^

^a–e^ Same letters within the same column are not significantly different according to Tukey’s post hoc test (*p* > 0.05). The mean ± standard deviation. ES: Eski Şirince Yolu, KK: Kınalı Köprü, AU: Abu Hayat Üst, AA: Abu Hayat Alt, and DB: Değirmen Boğazı.

**Table 2:** Phenolic profile of virgin olive oils from five different locations in the Selçuk region (*n* = 60, mg/kg as tyrosol).

		Groups				
	Phenolics (mg/kg)	ES	KK	AU	AA	DB
**Simple Phenols**	3,4-DHPEA (tyrosol)	1.55 ± 0.8 ^e^	3.89 ± 0.15 ^d^	11.53 ± 0.52 ^a^	2.36 ± 0.09 ^d^	7.28 ± 0.26 ^b^
	*p*-HPEA (hydroxytyrosol)	4.85 ± 0.14 ^e^	13.27 ± 0.55 ^b^	18.67 ± 0.84 ^a^	7.94 ± 0.38 ^d^	9.97 ± 0.42 ^c^
	*p*-coumaric acid	0.95 ± 0.03 ^d^	1.30 ± 0.07 ^a^	0.80 ± 0.03 ^e^	1.18 ± 0.04 ^b^	1.07 ± 0.05 ^c^
Lignan	Pinoresinol	18.31 ± 0.56 ^a^	15.61 ± 0.53 ^c^	16.63 ± 0.67 ^b^	14.01 ± 0.70 ^d^	13.06 ± 0.48 ^e^
Secoiridoids	3,4-DHPEA-EDA (oleacein)	45.19 ± 1.48 ^b^	30.69 ± 1.45 ^c^	45.37 ± 1.73 ^b^	6.87 ± 0.32 ^d^	55.95 ± 1.98 ^a^
	*p*-HPEA-EDA (oleocanthal)	93.58 ± 3.40 ^a^	82.34 ± 3.68 ^c^	86.92 ± 3.15 ^b^	42.43 ± 1.65 ^e^	73.55 ± 2.23 ^d^
	3,4-DHPEA-EA (oleuropein aglycone monoaldehyde)	40.02 ± 2.00 ^a^	25.24 ± 0.77 ^c^	40.44 ± 1.42 ^a^	26.63 ± 1.14 ^c^	32.60 ± 1.49 ^b^
	*p*-HPEA-EA (lignostride aglycone monoaldehyde)	15.21 ± 0.65 ^a^	11.05 ± 0.32 ^b^	6.52 ± 0.17 ^d^	6.50 ± 0.28 ^d^	8.64 ± 0.25 ^c^
	Total phenols	219.66	183.39	226.88	107.92	202.12

^a–e^ Same letters within the same row are not significantly different according to Tukey’s post hoc test (*p* > 0.05). The mean ± standard deviation. ES: Eski Şirince Yolu, KK: Kınalı Köprü, AU: Abu Hayat Üst, AA: Abu Hayat Alt, and DB: Değirmen Boğazı.

**Table 3:** Fatty acid composition of virgin olive oils from five different locations in the Selçuk region.

	Groups				
Fatty Acids (%)	ES	KK	AU	AA	DB
C16:0 (palmitic acid)	14.42 ± 0.4 ^a^	14.36 ± 0.72 ^a^	13.09 ± 0.66 ^ab^	13.86 ± 0.45 ^ab^	13.89 ± 0.58 ^b^
C16:1 (palmitoleic acid)	1.06 ± 0.02 ^b^	1.66 ± 0.03 ^a^	1.01 ± 0.02 ^c^	0.87 ± 0.02 ^d^	0.99 ± 0.02 ^c^
C17:0 (margaric acid)	0.04 ± 0.00 ^b^	0.08 ± 0.00 ^a^	0.04 ± 0.00 ^bc^	0.04 ± 0.01 ^bc^	0.035 ± 0.01 ^c^
C17:1 (margoleic acid)	0.06 ± 0.01 ^bc^	0.16 ± 0.01 ^a^	0.07 ± 0.00 ^b^	0.05 ± 0.00 ^d^	0.06 ± 0.01 ^cd^
C18:0 (stearic acid)	2.08 ± 0.09 ^b^	2.55 ± 0.09 ^a^	2.21 ± 0.10 ^b^	2.56 ± 0.07 ^a^	2.50 ± 0.13 ^a^
C18:1 (oleic acid)	68.18 ± 2.35 ^ab^	66.92 ± 2.35 ^b^	71.98 ± 3.18 ^a^	69.71 ± 3.47 ^ab^	69.24 ± 2.18 ^ab^
C18:2 (linoleic acid)	10.57 ± 0.52 ^a^	9.99 ± 0.36 ^a^	8.72 ± 0.36 ^b^	8.01 ± 0.35 ^c^	8.90 ± 0.39 ^b^
C20:0 (arachidic acid)	0.40 ± 0.01 ^b^	0.36 ± 0.01 ^c^	0.39 ± 0.01 ^b^	0.43 ± 0.01 ^a^	0.45 ± 0.01 ^a^
C18:3 (linolenic acid)	0.69 ± 0.01 ^a^	0.64 ± 0.01 ^b^	0.59 ± 0.02 ^c^	0.65 ± 0.01 ^b^	0.68 ± 0.02 ^a^
C20:1 (gadoleic acid)	0.30 ± 0.01 ^a^	0.24 ± 0.01 ^b^	0.30 ± 0.01 ^a^	0.29 ± 0.01 ^a^	0.30 ± 0.01 ^a^
C22:0 (behenic acid)	0.12 ± 0.00 ^bc^	0.10 ± 0.01 ^d^	0.11 ± 0.01 ^c^	0.13 ± 0.01 ^ab^	0.13 ± 0.01 ^a^
C24:0 (lignoseric acid)	0.07 ± 0.01 ^a^	0.05 ± 0.00 ^b^	0.06 ± 0.00 ^a^	0.07 ± 0.00 ^a^	0.07 ± 0.0 ^a^
Σ MUFA (monounsaturated fatty acids)	69.60 ± 2.34 ^a^	69.98 ± 2.35 ^a^	73.36 ± 3.17 ^a^	70.91 ± 3.17 ^a^	70.59 ± 2.18 ^a^
Σ PUFA (polyunsaturated fatty acids)	11.26 ± 0.51 ^a^	10.63 ± 0.36 ^a^	9.31 ± 0.38 ^bc^	8.66 ± 0.35 ^c^	9.58 ± 0.40 ^b^
Σ SAFA (saturated fatty acids)	17.12 ± 0.48 ^a^	17.50 ± 0.74 ^a^	15.90 ± 0.69 ^b^	17.08 ± 0.49 ^a^	17.07 ± 0.66 ^a^

^a–d^ Same letters within the same row are not significantly different according to Tukey’s post hoc test (*p* > 0.05). The mean ± standard deviation. ES: Eski Şirince Yolu, KK: Kınalı Köprü, AU: Abu Hayat Üst, AA: Abu Hayat Alt and DB: Değirmen Boğazı.

**Table 4:** Sterol composition of virgin olive oils from five locations in the Selçuk region (%).

	Groups				
Sterols (%)	ES	KK	AU	AA	DB
Cholesterol	0.14 ± 0.3	0.09 ± 0.01	0.15 ± 0.04	0.11 ± 0.03	0.10 ± 0.02
Brassicasterol	0.04 ± 0.01	0.07 ± 0.01	0.01 ± 0.00	0.02 ± 0.00	0.00 ± 0.00
24-methylene-cholesterol	0.04 ± 0.00	0.05 ± 0.01	0.08 ± 0.02	0.09 ± 0.01	0.07 ± 0.01
Campesterol	3.30 ± 0.12	3.34 ± 0.13	3.12 ± 0.19	3.13 ± 0.11	3.09 ± 0.11
Campestanol	0.37 ± 0.02	0.23 ± 0.01	0.35 ± 0.03	0.43 ± 0.02	0.27 ± 0.02
Stigmasterol	1.44 ± 0.08	1.36 ± 0.16	1.46 ± 0.09	1.67 ± 0.09	1.83 ± 0.07
Δ7-campesterol	0.04 ± 0.01	0.02 ± 0.00	0.01 ± 0.01	0.03 ± 0.01	0.02 ± 0.01
Clerosterol	0.99 ± 0.07	1.10 ± 0.05	1.26 ± 0.17	1.05 ± 0.17	1.04 ± 0.14
β-sitosterol	85.67 ± 0.22	86.92 ± 0.33	87.65 ± 0.39	85.99 ± 0.39	86.78 ± 0.26
Sitostanol	1.31 ± 0.07	1.62 ± 0.07	1.24 ± 0.08	1.82 ± 0.09	1.76 ± 0.08
Δ5-avenasterol	5.21 ± 0.10	3.92 ± 0.24	3.69 ± 0.34	4.08 ± 0.21	3.47 ± 0.15
Δ5.24-stigmastadienol	0.42 ± 0.02	0.48 ± 0.05	0.42 ± 0.04	0.44 ± 0.05	0.35 ± 0.07
Apparent β-sitosterol	93.59 ± 0.20	94.04 ± 0.26	94.27 ± 0.20	93.38 ± 0.24	93.39 ± 0.14
Δ7-stigmastenol	0.33 ± 0.03	0.32 ± 0.05	0.20 ± 0.03	0.40 ± 0.03	0.38 ± 0.03
Δ7-avenasterol	0.73 ± 0.05	0.47 ± 0.03	0.35 ± 0.04	0.74 ± 0.07	0.84 ± 0.08
Eritrodiol + uvaol	1.72 ± 0.28	1.29 ± 0.16	1.69 ± 0.20	1.78 ± 0.14	2.37 ± 0.54
Total Sterol	2086.08 ± 131.59	2059.93 ± 69.98	1155.43 ± 57.49	1109.23 ± 31.24	1364.30 ± 53.71

The mean ± standard deviation. ES: Eski Şirince Yolu, KK: Kınalı Köprü, AU: Abu Hayat Üst, AA: Abu Hayat Alt, and DB: Değirmen Boğazı.

## Data Availability

Data are contained within the article.
